# The Development of Novel Near-Infrared (NIR) Tetraarylazadipyrromethene Fluorescent Dyes

**DOI:** 10.3390/ma6051779

**Published:** 2013-05-06

**Authors:** Sung-Chan Lee, Duanting Zhai, Parag Mukherjee, Young-Tae Chang

**Affiliations:** 1 Laboratory of Bioimaging Probe Development, Singapore Bioimaging Consortium, Agency for Science, Technology and Research (A*STAR), Biopolis, 138667, Singapore; E-Mail: lee_sung_chan@sbic.a-star.edu.sg; 2 Department of Chemistry, Medicinal Chemistry Program of Life Sciences Institute, National University of Singapore, 3 Science Drive 3, 117543, Singapore; E-Mail: g0800736@nus.edu.sg (D.Z.); paragmcy@gmail.com (P.M.)

**Keywords:** Near Infra-Red, fluorescent, aza-BODIPY

## Abstract

Novel structures of an near-infrared (NIR) tetraarylazadipyrromethene (aza-BODIPY) series have been prepared. We designed the core structure containing two amido groups at the para-position of the aromatic rings. The amido group was incorporated to secure insensitivity to pH and to ensure a bathochromic shift to the NIR region. Forty members of aza-BODIPY compounds were synthesized by substitution of the acetyl group with commercial amines on the alpha bromide. The physicochemical properties and photostability were investigated and the fluorescence emission maxima (745~755 nm) were found to be in the near infrared (NIR) range of fluorescence.

## 1. Introduction

There is an imperative need for the development of new and effective dyes that emit fluorescence in the near-infrared (NIR) region (*i.e.*, 700~1000 nm) for *in vivo* biological applications [1,2,3,4]. Fluorophores having absorption/emission profiles in the NIR regions are very useful, due to deep penetration of the NIR light, significant reduction of the background signal, low light scattering, as well as being a cheap illumination light source. So far, several scaffolds have been developed as NIR fluorophores such as squarine, quinone and cyanine. As well as having the most extensively developed NIR dye, our research group has also reported synthesis of novel cyanine libraries and their application for fluorescence and SERS *in vivo* animal imaging [5,6,7,8,9]. NIR dyes with good photostability have been identified, especially from the cyanine libraries, which would benefit *in vivo* bioimaging.

Since O’Shea and co-workers reported the interesting structures of NIR aza-BODIPY and studied their spectral properties [10], aza-BODIPY compounds had only been prepared in an *ad hoc* manner, which limited their potential as NIR imaging probes [11]. The diversity-oriented fluorescence library approach (DOFLA) provides a convenient way to generate a large number of fluorescent libraries [12]. Based on previous studies, we intend to construct a fluorescent library in theNIR region, utilizing the superior photophysical properties of aza-BODIPY compounds. Here, we report the synthesis and physicochemical characterization of novel aza-BODIPY NIR derivatives.

## 2. Experimental Section

### 2.1. Materials & Method

All reactions were performed in oven-dried glassware under a positive pressure of nitrogen. Unless otherwise noted, starting materials and solvents were purchased from Aldrich and Acros organics and used without further purification. Analytical TLC was carried out on Merck 60 F254 silica gel plate (0.25 mm layer thickness) and visualization was done with UV light. Column chromatography was performed on Merck 60 silica gel (230–400 mesh). NMR spectra were recorded on a Bruker Avance 300 MHz NMR spectrometer. Chemical shifts are reported as δ in units of parts per million (ppm) and coupling constants are reported as a J value in Hertz (Hz). Masses of all the compounds were determined by LC-MS of Agilent Technologies with an electrospray ionization source. The Phenomenex C18 (50 mm × 4.6 mm, 5 µm) column was used for purity confirmation with 0.1% TFA containing acetonitrile/water gradient (5% to 100% in 10 min) elution conditions. Spectroscopic measurements were performed on a fluorometer and a UV/Vis instrument, Synergy 4 of bioteck company and Gemini XS fluorescence plate reader. The slit width was 1 nm for both excitation and emission. Relative quantum efficiencies were obtained by comparing the areas under the corrected emission spectra. The following equation was used to calculate quantum yield.

*Φ_x_* = *Φ_st_*(*I_x_*/*I_st_*)(*A_st_*/*A_x_*)(*η_x_*^2^/*η_st_*^2^)

where *Φ_st_* is the reported quantum yield of the standard, *I* is the integrated emission spectrum, *A* is the absorbance at the excitation wavelength, and *η* is the refractive index of the solvents used. The subscript × denotes unknown and *st* denotes the standard. Tetraphenyl-aza-bodipy was used as standard.

Photobleaching experiments in DMSO solutions were performed by irradiating the samples with light from a 365 nm high-power 30 mW, UV lamp. The photodegradation profiles were obtained by monitoring the fluorescence spectra.

### 2.2. Synthesis

Synthesis of Compound 2. To an EtOH solution (100 mL) of the *p*-Boc-aminoacetophenone (1 g, 4.25 mmol) and benzaldehyde (1 equiv), 4 M NaOH (0.5 mL) was added. The mixture solution was heated at 60 °C for 20 h. After cooling to room temperature, the solvent was removed *in vacuo*, and the oily residue obtained was neutralized with 1 M HCl and partitioned between EtOAc (250 mL) and H_2_O (250 mL). The organic layer was separated, dried over sodium sulfate, and evaporated under reduced pressure. The obtained product was purified on silica with EtOAc and Hexane (1.2 g, 87%).

^1^H NMR (300 MHz, CDCl_3_) δ 8.01 (d, *J* = 8.7 Hz, 2H), 7.80 (d, *J* = 15.6 Hz, 1H), 7.66–7.63 (m, 2H), 7.56–7.39 (m, 6H), 6.77 (s, 1H), 1.53 (s, 9H); ^13^C NMR (75 MHz, CDCl_3_) δ 188.8, 152.1, 144.2, 142.7, 135.0, 132.7, 130.4, 130.1, 128.9, 128.4, 121.8, 117.5, 81.3, 28.3; ESI-MS *m*/*z* (M + H) calcd: 324.2, found 324.2.

Synthesis of Compound 3. A solution of chalcone (1 g, 3.1 mmol), nitromethane (20 equiv) and KOH (0.2 equiv) in EtOH (100 mL) was heated at 60 °C for 12 h. After cooling to room temperature, the solvent was removed *in vacuo*, and the residue was partitioned between EtOAc (150 mL) and H_2_O (150 mL). The organic layer was separated, dried over sodium sulfate, and evaporated under reduced pressure. The obtained product was purified by column chromatography (1.1 g, quantitative).

^1^H NMR (300 MHz, CDCl_3_) δ 7.90 (d, *J* = 8.73 Hz, 2H), 7.48 (d, *J* = 8.73 Hz, 2H), 7.37–7.30 (m, 5H), 6.79 (s, 1H), 4.87 (dd, *J* = 6.4, 12.4 Hz, 1H), 4.71 (dd, *J* = 8.1, 12.4, 1H), 4.27–4.22 (m, 1H), 3.45–3.40 (m, 2H), 1.56 (s, 9H); ^13^C NMR (75 MHz, CDCl_3_) δ 195.3, 152.0, 143.3, 139.1, 130.8, 129.5, 128.9, 127.8, 127.4, 117.4, 81.4, 79.5, 41.1, 39.3, 28.2; ESI-MS *m*/*z* (M + H) calcd: 385.2, found 385.2.

Synthesis of Compound 4. Compound 3 (1 g, 2.6 mmol), and ammonium acetate (35 equiv) in 1-butanol (150 mL) were heated at 120 °C for 6 h. The reaction was cooled to room temperature, solvent was removed *in vacuo* and the crude product was purified by column chromatography on silica-gel eluting with 1% MeOH in CH_2_Cl_2_ to yield compound 4 as blue-black solid.

^1^H NMR (300 MHz, DMSO-d_6_) δ 9.79 (s, 2 H), 8.09 (d, *J* = 7.23 Hz, 4H), 8.00 (d, *J* = 8.7 Hz, 4H), 7.72 (d, *J* = 8.7 Hz, 4H), 7.59 (s, 2H), 7.49–7.38 (m, 6H), 1.53 (s, 18H); ESI-MS *m*/*z* (M + H) calcd: 680.3, found 680.2.

Synthesis of Compound 5. Compound 4 (0.3 g, 0.44 mmol) was dissolved in dry CH_2_Cl_2_ (50 mL), treated with DIEA (10 equiv) and BF_3_ diethyletherate (15 equiv), and stirred under nitrogen for 24 h. The reaction mixture was quenched with sat. NaHCO_3_ solution (10 mL). The organic layer was separated, dried over sodium sulfate, and evaporated under reduced pressure. Purification by column chromatography on silica eluting with 1%~2% of MeOH in CH_2_Cl_2_ yielded the product as a dark blue solid.

^1^H NMR (300 MHz, DMSO-d_6_) δ 8.16 (d, *J* = 7.4 Hz, 4H), 8.05 (d, *J* = 8.7 Hz, 4H), 7.54–7.42 (m, 8H), 6.71 (d, *J* = 8.8 Hz, 4H), 6.35 (s, 4H); ESI-MS *m*/*z* (M + H) calcd: 528.2, found 528.2.

Synthesis of Compound 6. Compound 5 (0.2 g, 0.38 mmol) was dissolved in acetonitrile (100 mL), sat. NaHCO_3_ solution (1 mL) and bromoacetyl bromide (5 equiv) were added and stirred for 10 min. The solvent was removed *in vacuo*, and partitioned between EtOAc (250 mL) and saturated NaHCO_3_ solution (100 mL). The organic layer was separated, dried over sodium sulfate, and evaporated under reduced pressure. The obtained product was used for further synthesis without purification.

^1^H NMR (300 MHz, DMSO-d_6_) δ 10.8 (s, 2H), 8.20–8.07 (m, 8H), 7.86–7.78 (m, 4H), 7.64–7.39 (m, 8H), 4.34 (s, 4H); ESI-MS *m*/*z* (M + Na) calcd: 791.0, found 790.8.

General synthetic procedure for AZA derivatives; A solution of compound 6 (20 mg, 26 µmol), DIEA (5 equiv) and building block amine (5 equiv) was stirred for 5 h at room temperature. The solvent of the reaction mixture was removed *in vacuo*, and the residue was partitioned between EtOAc (5 mL) and H_2_O (5 mL). The separated organic layer was washed with saturated citric acid solution and sat. NaHCO_3_ solution. The crude product was purified by preparative thin layer column chromatography on silica eluting with 10% MeOH in CH_2_Cl_2_ to yield the final compounds (see supporting information; Table S1 for the summary of properties and Figure S1 for the representative LC-MS data).

Characteristics of AZA396; AZA396 was synthesized according to the general synthetic procedure. ^1^H-NMR (DMSO-d_6_) δ 8.16 (m, 8H), 7.86 (m, 5H, ), 7.54 (m, 9H), 7.32 (d, *J* = 7.9, 5H), 7.17 (d, *J* = 7.9, 5H), 3.86 (s, 4H), 2.50 (s, 4H), 2.29 (s, 6H); ^13^C-NMR (DMSO-d_6_) δ 168.95, 168.90, 136.60, 134.43, 129.02, 128.91, 128.74, 128.62, 51.46, 20.73; ESI-MS *m*/*z* (M + H) calcd: 850.4, found 850.4.

## 3. Results and Discussion

### 3.1. Synthesis

**Figure 1:**
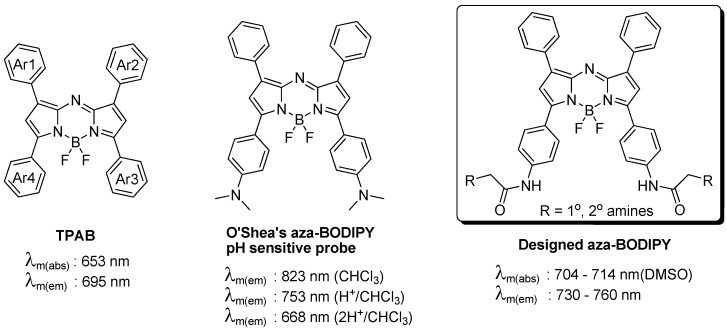
Tetraarylazadipyrromethene (aza-BODIPY) structures and the new designed scaffold.

**Scheme 1:**
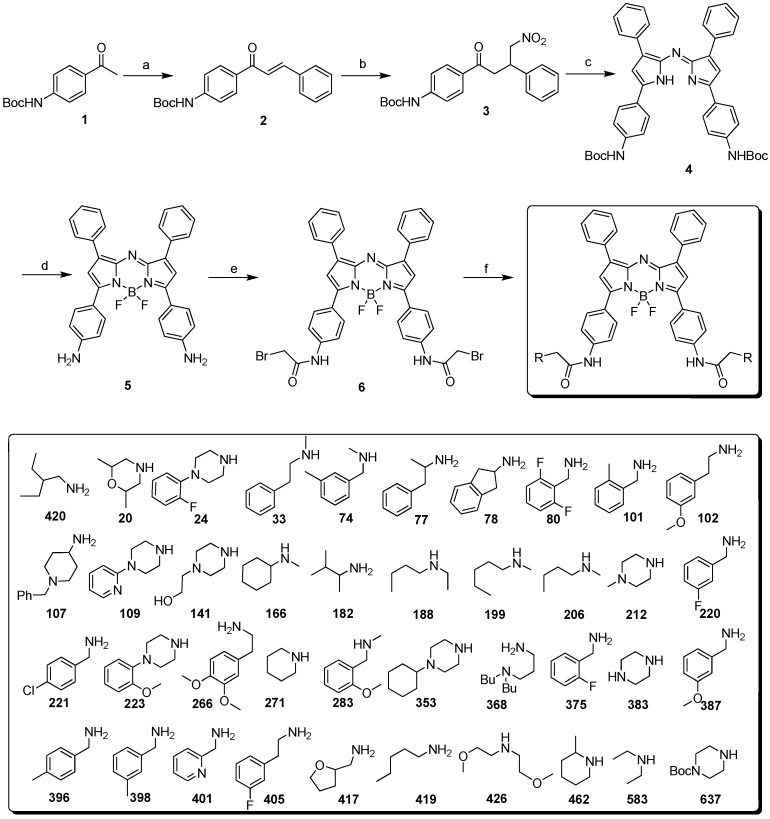
(**a**) Benzaldehyde, KOH; EtOH, 60 °C (**b**) nitromethane, diethylamine or KOH, EtOH, 60 °C; (**c**) ammonium acetate, n-BuOH, reflux (**d**) BF_3_-etherate, DIEA; in DCM (**e**) BrAcCl, acetonitrile, saturated aqueous NaHCO_3_ (**f**) 1° and 2° amines, DIEA, DMF, RT.

The tetraphenyl-substituted aza-BODIPY (**TPAB**) chromophore has maximum fluorescence emission at 695 nm which is slightly shorter than the NIR region. One of the reported NIR aza-BODIPY scaffolds is O’Shea’s amine containing structure. It has a large *bathochromic shift* by conjugation of n→π* of the lone pair of electrons of the amine, however this amine also affects both the quantum yield and the pH sensitive properties. In order to develop a new NIR aza-BODIPY having both low pH sensitivity and good physicochemical properties, we designed an amido substituted aza-BODIPY (Figure 1). The n→π* conjugation can be partially removed by the resonance effect of the amide bond and the moderate *hypsochromic shift* can be achieved from O’Shea’s structure. The synthesis of aza-BODIPY started from the diaryl α,β-unsaturated ketone (chalcone, **2**), which was prepared by the Claisen–Schmidt condensation using benzaldehyde and Boc protected 4′-aminoacetophenone (**1**) with NaOH as a base. Michael addition of nitromethane to the chalcone yielded the 1,3-diaryl-4-nitrobutan-1-one (**3**) with diethylamine or KOH as base in almost quantitative yield after aqueous workup and this intermediate was then used for the next step without further purification. Condensation with ammonium acetate in refluxing butanol rendered azadipyrromethene (**4**) via a cascade event where pyrrole and the corresponding nitrosopyrrole were formed *in situ* following subsequent condensation. The obtained mixture was purified by chromatography with hexane/dichloromethane. Subsequent complexation of the azadipyrromethene with boron trifluoride gave the aza-BODIPY (**5**) in good yield. Simultaneously, Boc deprotection was completed by excess BF_3 _etherate. The key intermediate **6** was prepared by bromoacetylation. As a final step, 40 aza-BODIPY compounds (named as **AZA**) were synthesized by substitution of the bromide with commercial amines at room temperature (Scheme 1).

**Scheme 2:**
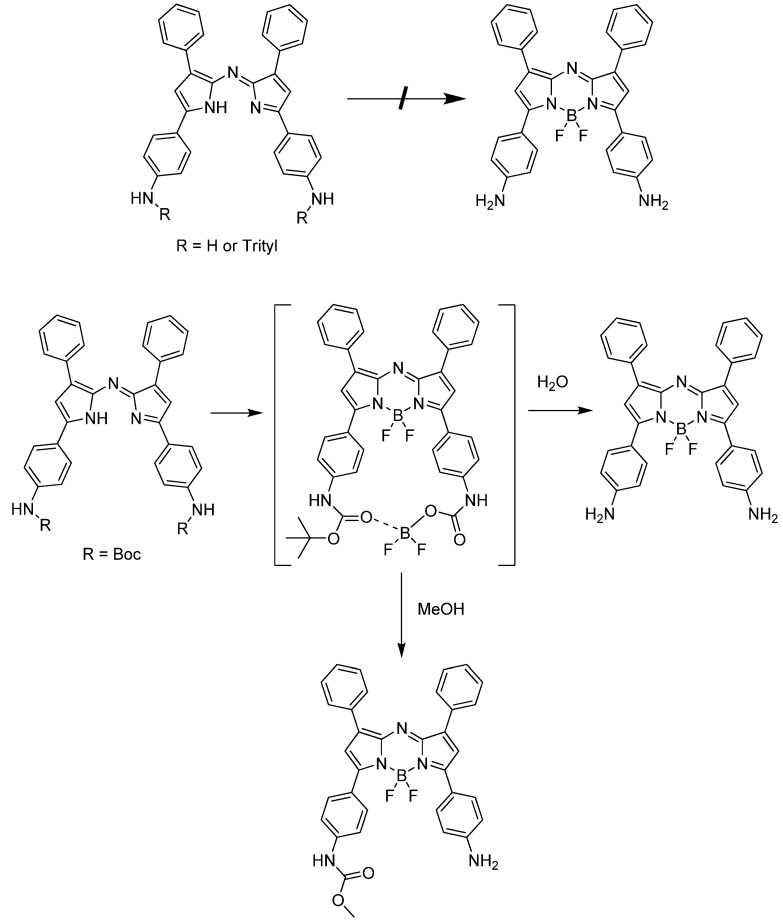
Modified test route for the preparation of aza-BODIPY.

An interesting result was observed during the optimization of Scheme 1. Without the protection of the amino group in compound **1** or with other protective groups such as triphenylmethyl, each intermediate step could be prepared successfully, except for the BF_3_ complexed product (Scheme 2). This may be explained by steric interference of the trityl group [13]. However, the steric interaction cannot explain the case of free amino and Boc-amino-azadipyrromethene. Presumably, Boc is coordinated by boron as an unstable cyclic intermediate and this observation can be supported by the fact that when step **d** in Scheme 1 was quenched by methanol, a methylcarbamate product was obtained on one side of the amine (Scheme 2).

### 3.2. Spectroscopic Properties

**Figure 2:**
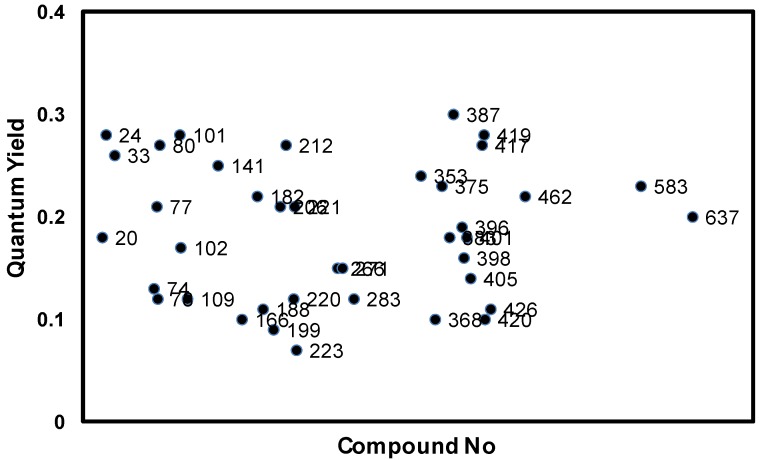
Quantum yield distribution of **AZA** compounds; quantum yields (QYs) are measured in 10 μM DMSO solution and calculated using tetraphenyl-substituted aza-BODIPY (**TPAB**) as reference. Numbers correspond to the amine numbers in Scheme 1.

The new **AZA** compounds were dissolved in dimethylsufoxide, and their spectroscopic properties were studied. The wavelengths of the absorption maxima are observed in the range of 704 to 714 nm, while the fluorescence emission maxima are in the range of 745 to 755 nm. The fluorescence quantum yields (QYs) of the dyes are also distributed in a similar range (0.1~0.3) (Figure 2).

**Figure 3:**
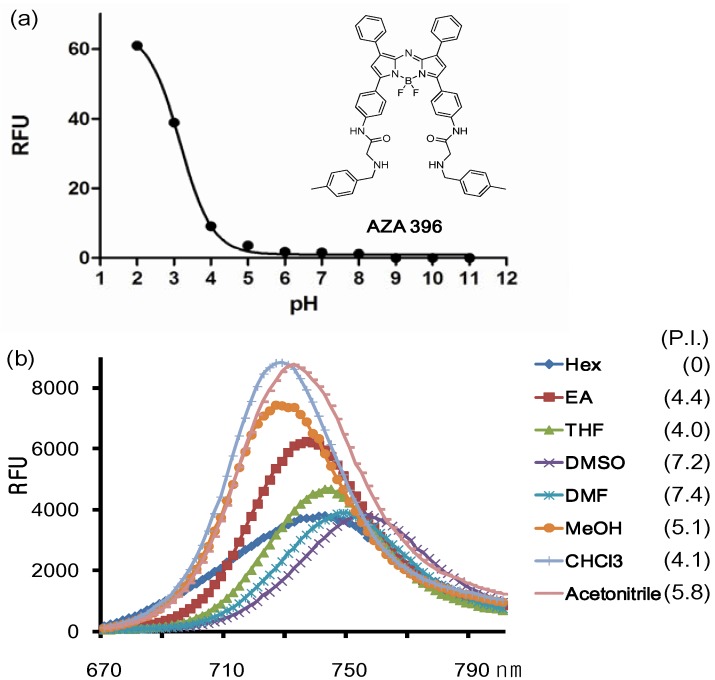
(**a**) Fluorescence changes of the representative compound **AZA**396 with pH and (**b**) solvents (numbers are polarity index values).

We picked out one representative compound named **AZA**396 (λ_abs_: 707 nm; λ_flu_: 754 nm; Quantum yield: 0.19; ε: 909925 M^−1^ cm^−1^) and studied its properties further (Figure 3).

The conjugation of the Ar3 and Ar4 rings of the aza-BODIPY chromophore with the amido group renders a dramatic bathochromic shift which is located in the middle of the values of TPAB and O’Shea’s aza-BODIPY shown in Figure 1. The emission maximum of the probe was affected by solvent and polarity. The maximum emission peaks were distributed in a small range of 25 nm and also the full width at half the maximum peak height had almost the same value (40 nm). Interestingly, the intensity was slightly different depending on solvent but there was no clear connection between intensity and solvent properties except for the dramatic difference between organic solvent and in aqueous media (based on comparison between Figure 3a and Figure 3b; F(organic solvents)/F(aqueous media) > 1000). In aqueous solution, due to the high lipophilicity, **AZA**396 exhibited almost no emission intensity at high pH, but its fluorescent intensity was slightly turned on at low pH (pH = 2 ~ 4) without a bathochromic shift. Presumably, the photoinduced electron transfer (PET) effect of the amido group did not change at low pH and dye disaggregation was induced by protonation of the secondary amines.

### 3.3. Photostability

**Figure 4:**
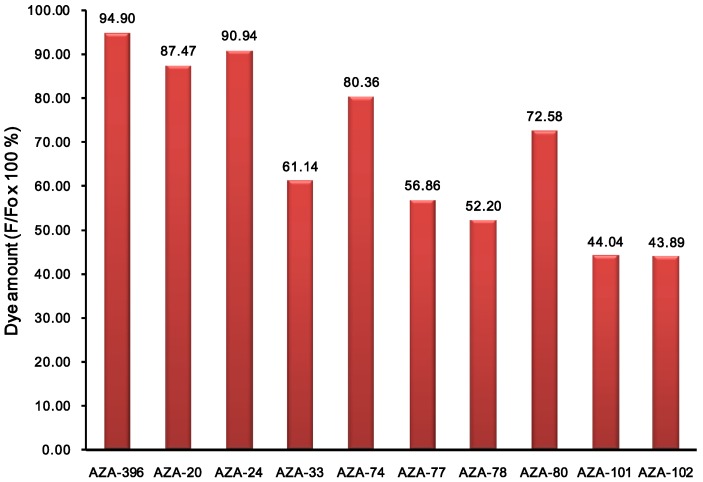
Photostability test of representative compounds in DMSO solution (at 50 μM). Photostability was measured by fluorescence intensity (F) retention at 80 min under irradiation of UV with a 30 mW lamp at 365 nm. Fo is the initial fluorescent intensity. Compound ID numbers correspond to the building block amines.

**Figure 5:**
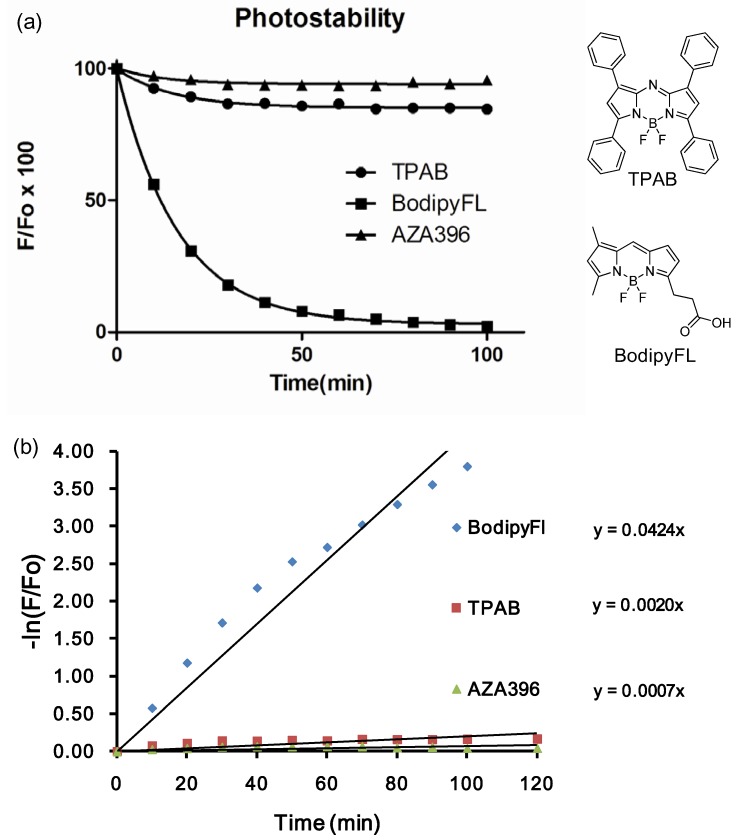
(**a**) Photostability test of **AZA**396 compare with standards compounds tetraphenyl aza-bodipy (TPAB) and BodipyFl acid in DMSO; (**b**) Values are fitted to a non-linear regression one-phase exponential decay. Rate constants of dye photodecomposition were acquired from the plots of −ln(F/F^0^) *vs.* time, considering ln(F/F^0^) = k*t as a pseudo-first order rate equation.

We further investigated the photostability of the dyes in dimethylsulfoxide under continuous illumination with a high power UV lamp at 365 nm. The photodegradation profiles for 10 representative dyes are shown in Figure 4. Photodegradation was measured by the difference in fluorescence intensity before and after 80 min irradiation. In fact, after 80 min of illumination, most of the dyes were still emitting significant fluorescence emission. Average degradation percentage was 32% (lowest 5% and highest 56%). The most photostable compound, **AZA**396 was further compared with two control compounds (*i.e.*, TPAB and BodipyFl as positive and negative references) by measuring their photodegradation at 10 min intervals (Figure 5).

To compare the degradation rate between **AZA**396 and control compounds, values were fitted to a non-linear regression. About 60-fold rate constant differences between BodipyFl and **AZA**396 reflected the superior photostability of the **AZA** compound making, it particularly suitable for long-duration measurements (Figure 5b).

## 4. Conclusions

In summary, novel structures of an aza-BODIPY series have been prepared and developed as NIR imaging probes. The photophysical properties of the new aza-BODIPY derivatives were evaluated and showed high photostability and acceptable fluorescence quantum yields.

Specifically, **AZA**396 can be developed with the potential to be exploited and adapted to suit a diverse range of NIR bio-imaging applications.

## Supplementary Materials

Supplementary File 1
