# Supplementary material for: The Development of Novel Near-Infrared (NIR) Tetraarylazadipyrromethene Fluorescent Dyes
Source: Materials (Basel). 2013 May 6;6(5):1779–88. doi: 10.3390/ma6051779 (PMC5452499; doi:10.3390/ma6051779)
Supplement: Supplementary File 1 [file materials-06-01779-s001.pdf]

Supporting Information

Table S1. Spectroscopic properties and identification data of AZA compounds. *Purity was calculated at 254 nm and some of the impurity was identified as decomposed (-BF2) byproduct with no effect on QY.

ID	Abs (nm)	Em (nm)	M.W.	Mass$[M+H]^+$	QY	Purity* (%)
AZA-420	703	747	839.5	840.5	0.10	80
AZA-20	707	756	837.8	838.4	0.18	79
AZA-24	703	751	967.9	484.8:$[M+2H]^{2+}$	0.28	85
AZA-33	703	749	877.8	878.8	0.26	88
AZA-74	710	755	877.8	878.9	0.13	86
AZA-77	706	753	877.8	878.9	0.21	78
AZA-78	707	755	873.3	874.2	0.12	90
AZA-80	707	756	893.7	447.8:$[M+2H]^{2+}$	0.27	92
AZA-101	707	755	849.8	850.4	0.28	88
AZA-102	710	756	909.8	910.4	0.17	86
AZA-107	703	745	988.0	988.8	0.30	78
AZA-109	710	748	933.9	467.8:$[M+2H]^{2+}$	0.12	87
AZA-141	707	753	867.8	868.4	0.25	89
AZA-166	707	754	833.8	834.4	0.10	77
AZA-182	707	749	781.7	782.4	0.22	91
AZA-188	706	752	809.8	810.4	0.11	91
AZA-199	710	753	809.8	810.4	0.09	86
AZA-206	704	748	781.7	782.4	0.21	88
AZA-212	708	753	807.7	808.4	0.27	87
AZA-220	703	757	857.3	858.3	0.12	97
AZA-221	714	759	890.6	891.4	0.21	78
AZA-223	714	745	991.9	496.9:$[M+2H]^{2+}$	0.07	79
AZA-266	707	746	969.9	970.6	0.15	88
AZA-271	708	757	777.7	778.4	0.15	65
AZA-283	711	759	909.8	910.5	0.12	59
AZA-353	710	759	944.0	472.9:$[M+2H]^{2+}$	0.24	79
AZA-368	707	747	1096.3	1097.5	0.10	90
AZA-375	711	756	857.7	858.6	0.23	86
AZA-383	705	743	779.7	780.8	0.18	88
AZA-387	702	743	881.8	882.4	0.34	75
AZA-396	707	754	849.8	850.4	0.19	91
AZA-398	710	755	849.8	850.3	0.16	87
AZA-401	703	748	823.7	824.4	0.18	69
AZA-405	705	754	885.8	886.4	0.14	73
AZA-417	703	744	809.7	810.3	0.27	86
AZA-419	707	737	781.4	781.2	0.28	85
AZA-426	708	752	873.8	874.8	0.11	89
AZA-462	704	745	805.8	806.4	0.22	94
AZA-583	703	742	753.7	754.4	0.23	87
AZA-637	710	752	979.9	980.6	0.20	85

Figure S1. Representative LC-MS chromatogram.

AZA 20

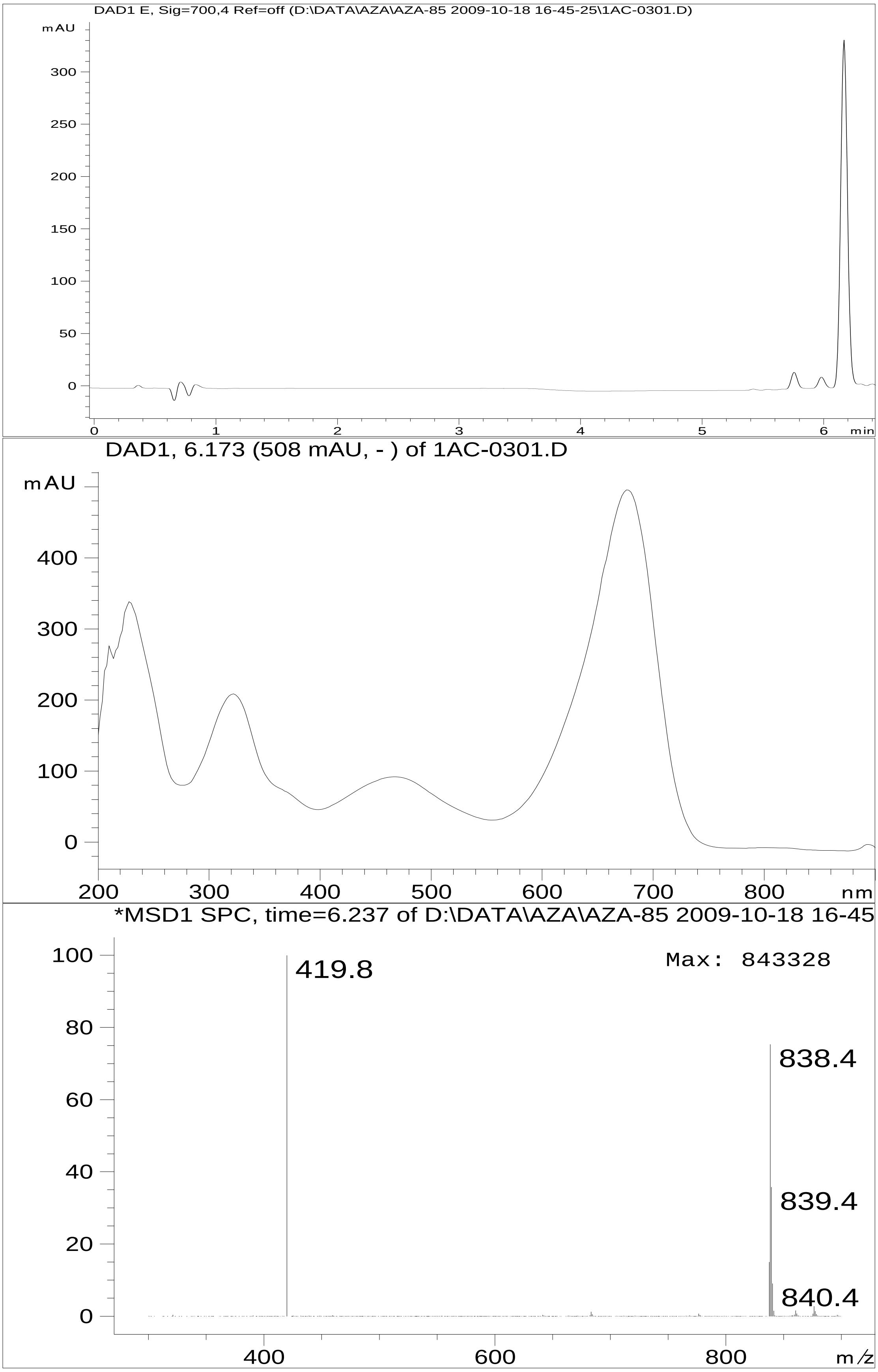

Figure S1. *Cont.*

AZA 24

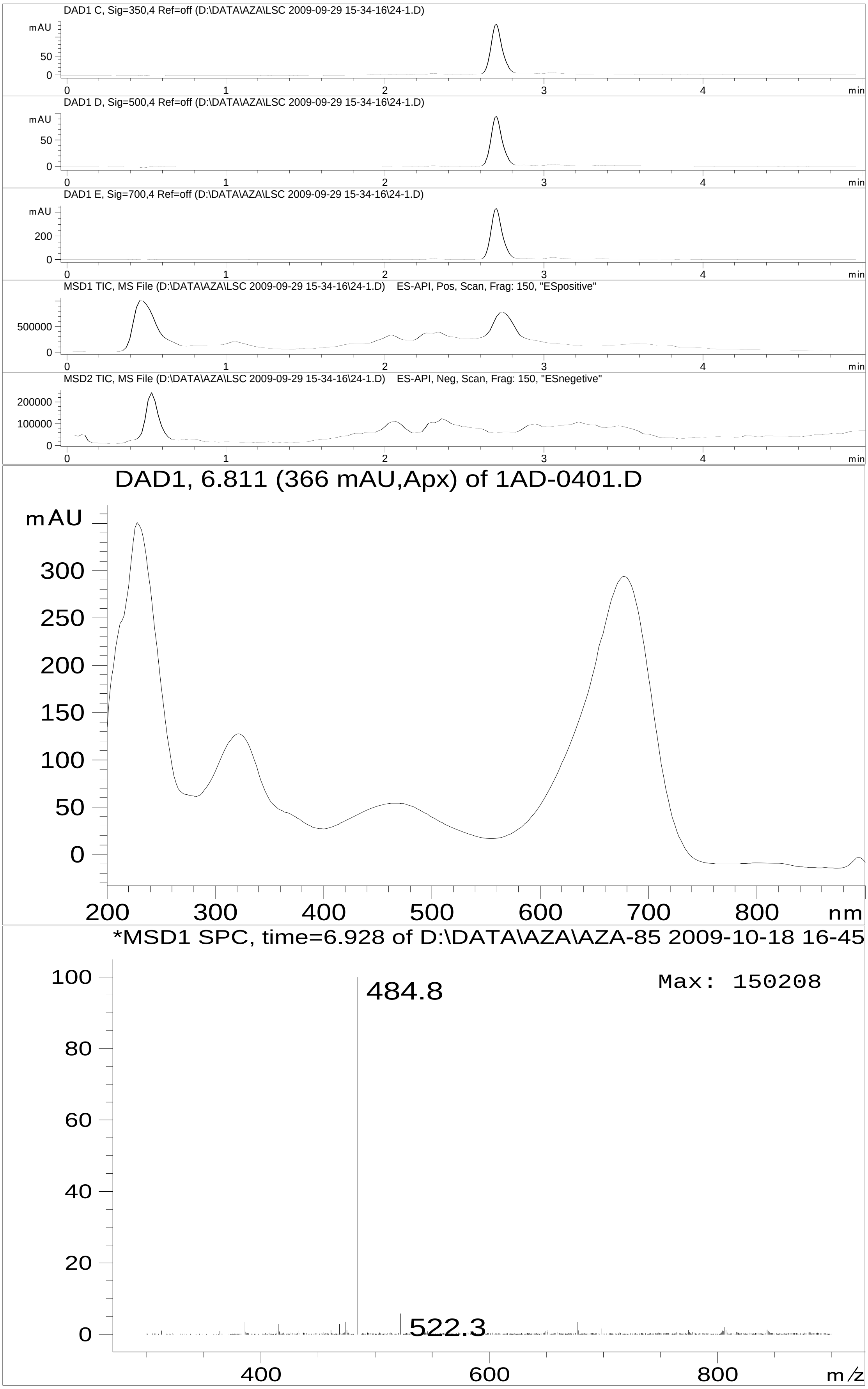

Figure S1. *Cont.*

AZA 33

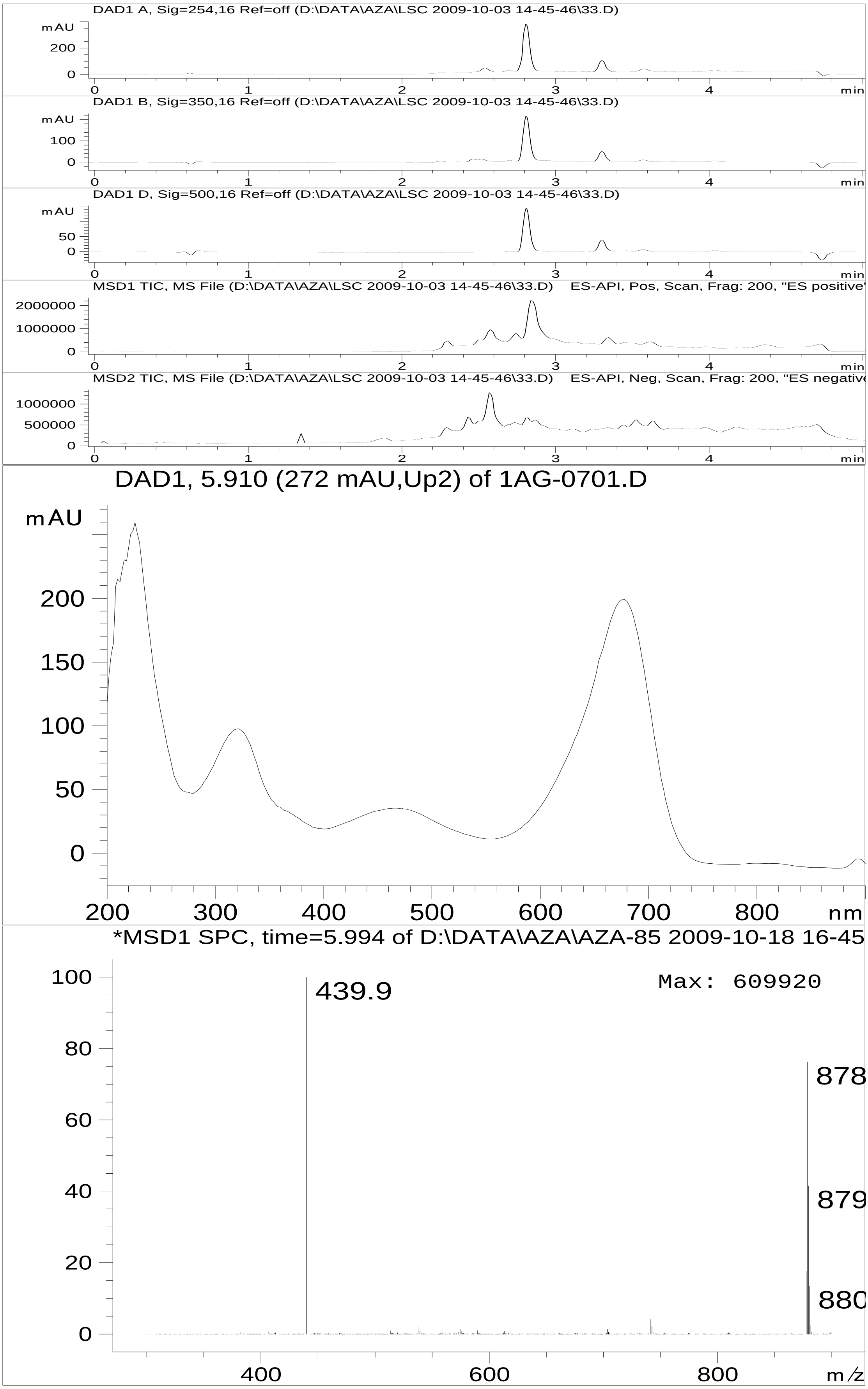

Figure S1. *Cont.*

AZA 74

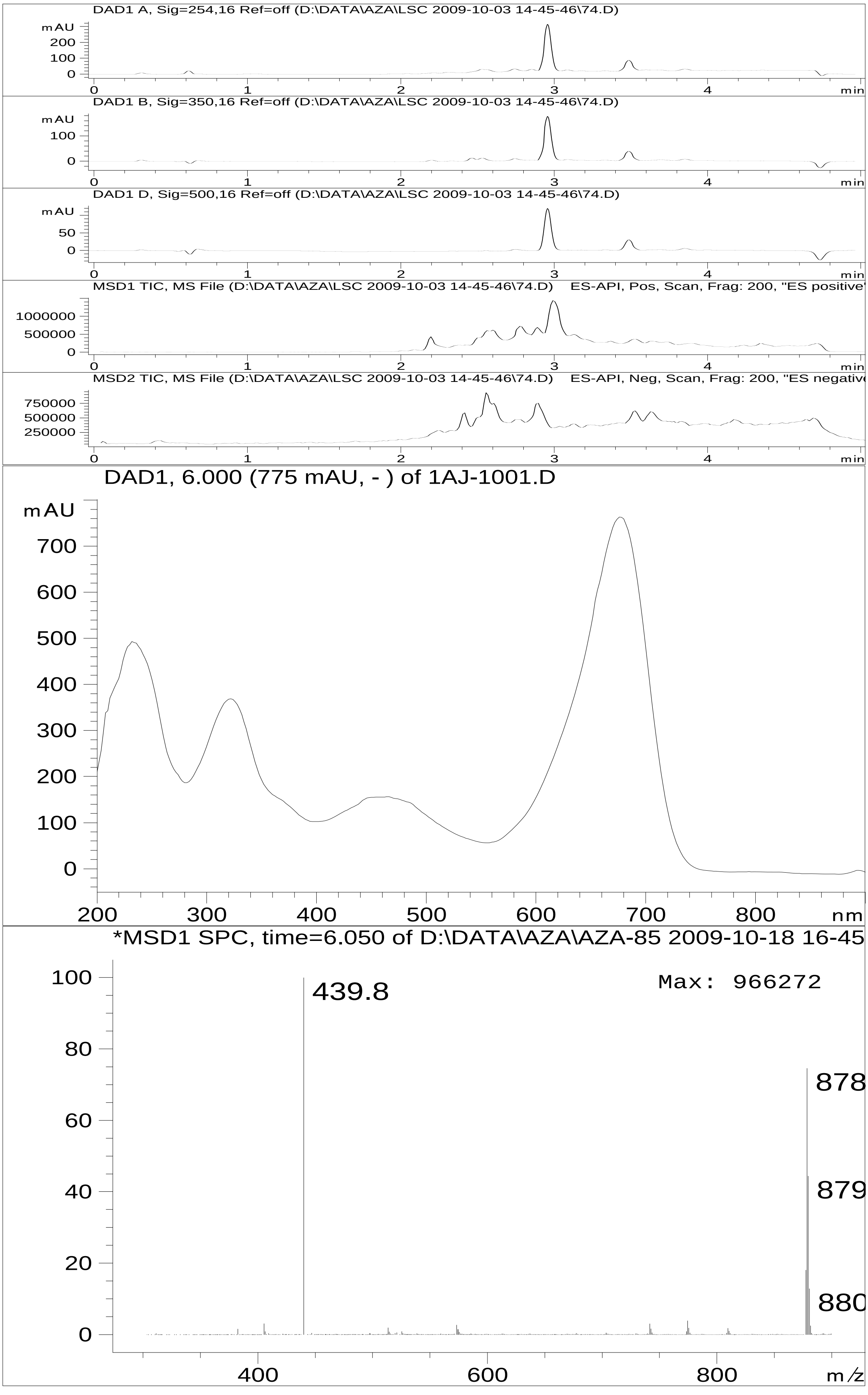

Figure S1. *Cont.*

AZA 77

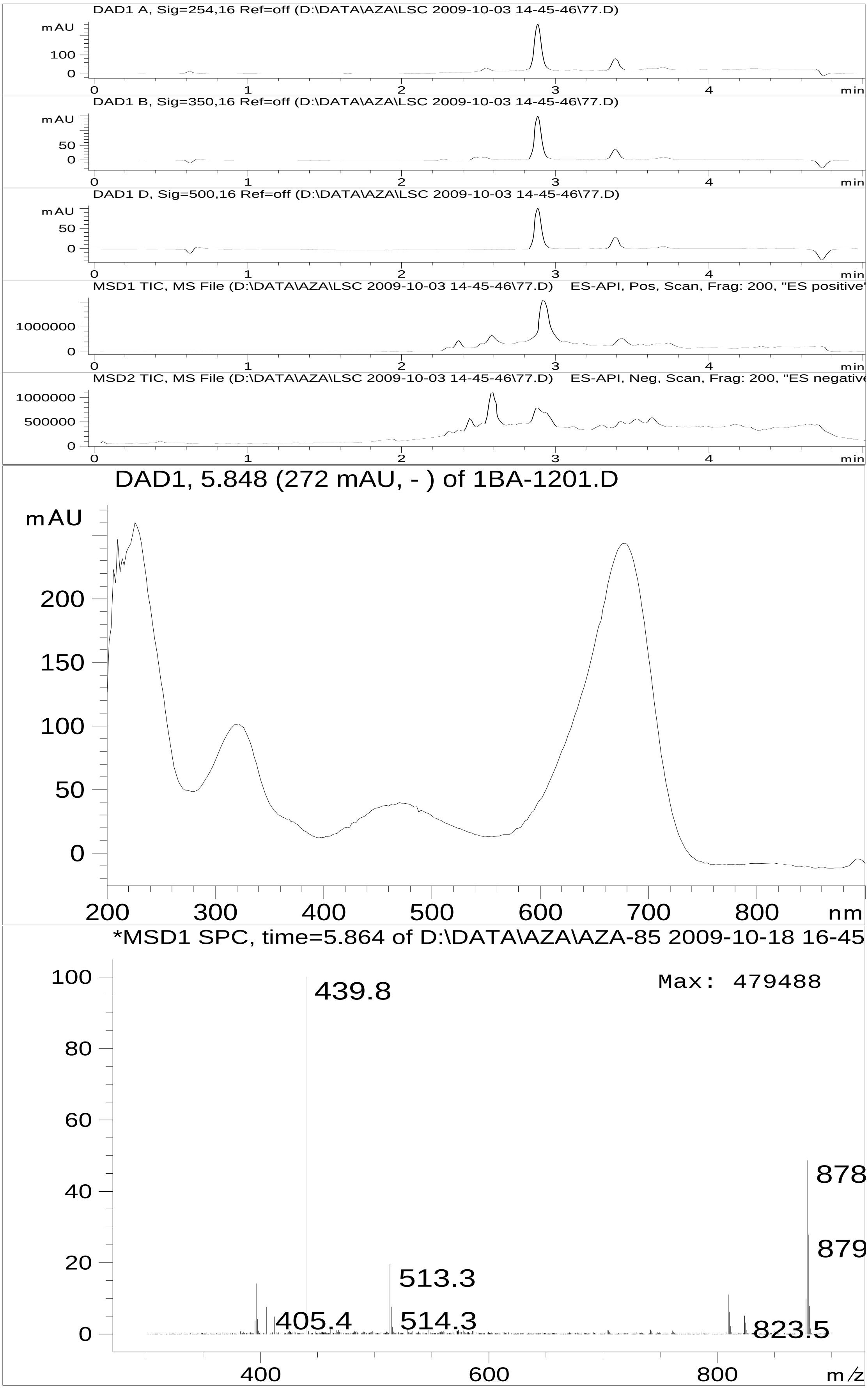

Figure S1. *Cont.*

AZA 80

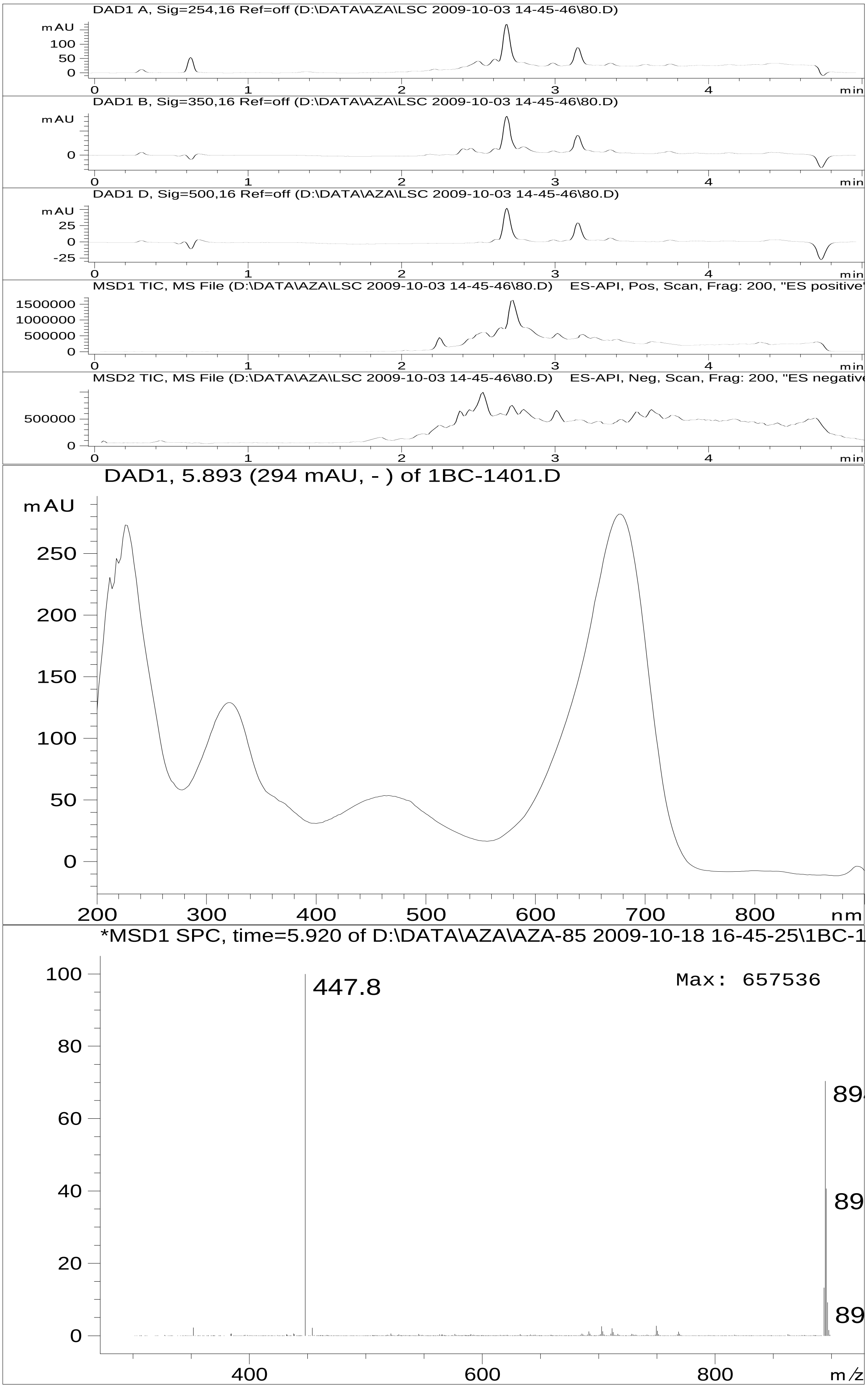

Figure S1. *Cont.*

AZA 206

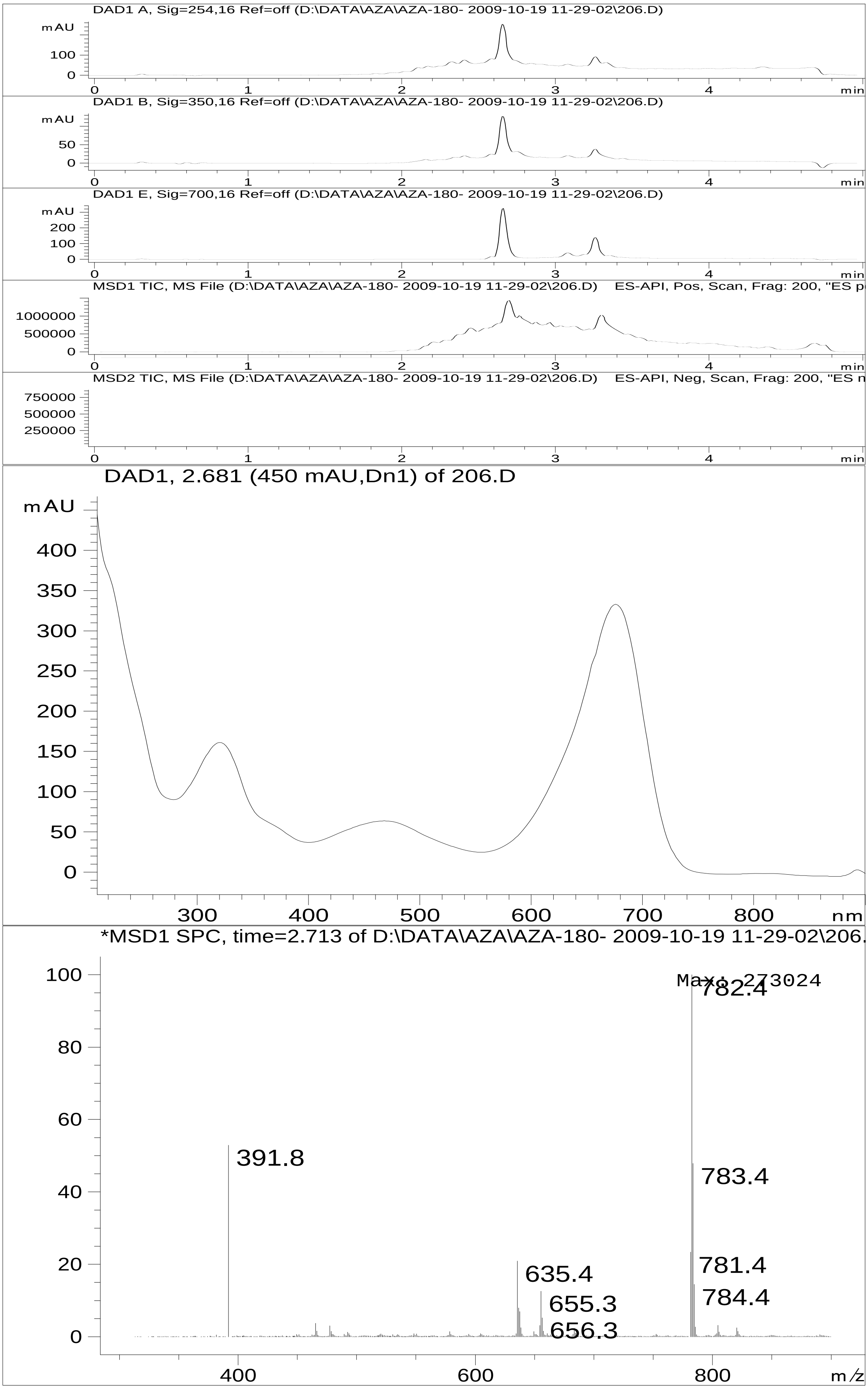

Figure S1. *Cont.*

AZA 212

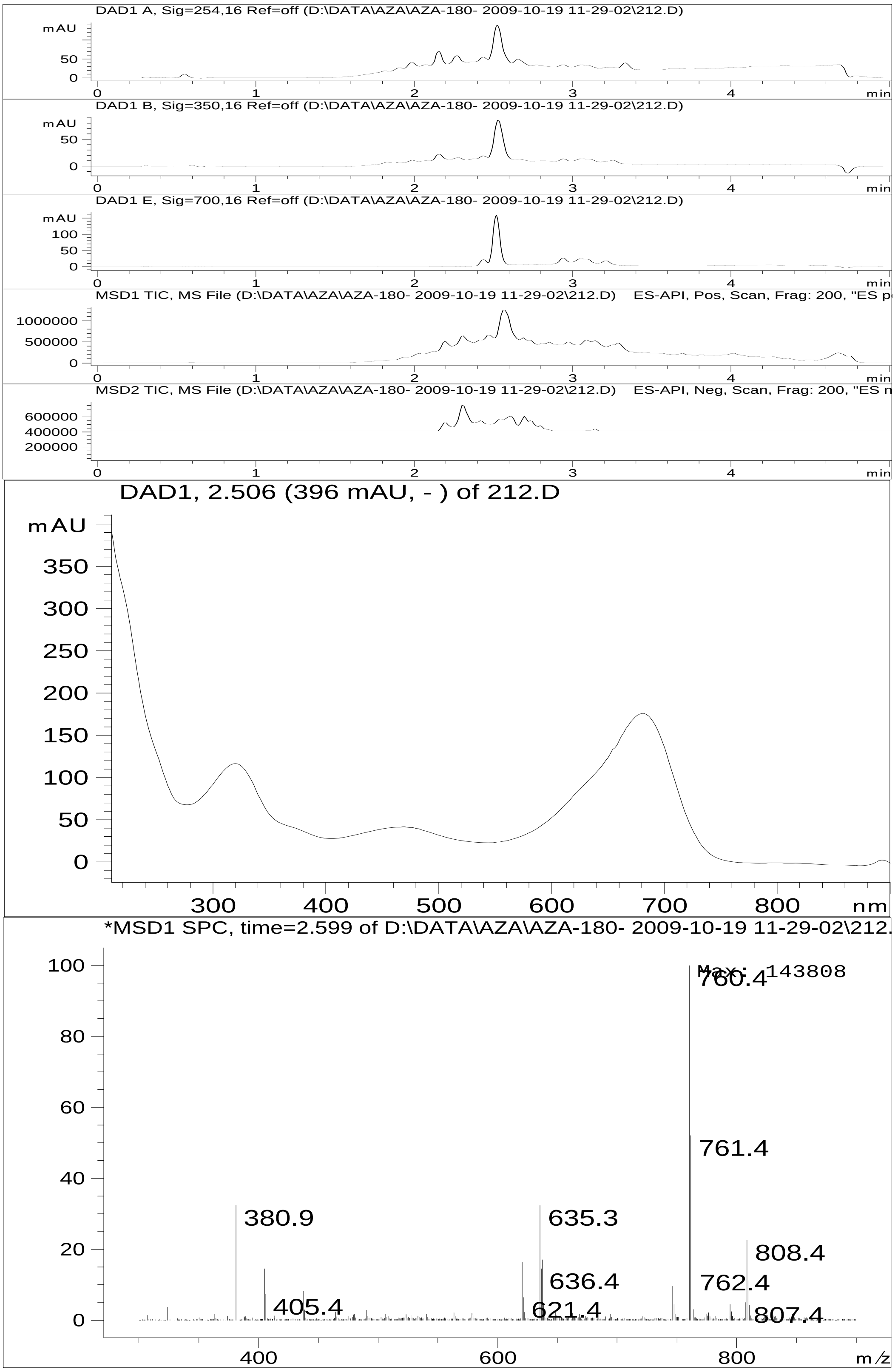

© 2013 by the authors; licensee MDPI, Basel, Switzerland. This article is an open access article distributed under the terms and conditions of the Creative Commons Attribution license (http://creativecommons.org/licenses/by/3.0/).
